# Determining HIV risk for Adolescent Girls and Young Women (AGYW) in relationships with “Blessers” and age-disparate partners: a cross-sectional survey in four districts in South Africa

**DOI:** 10.1186/s12889-022-13394-4

**Published:** 2022-05-14

**Authors:** Gavin George, Sean Beckett, Tarylee Reddy, Kaymarlin Govender, Cherie Cawood, David Khanyile, Ayesha B. M. Kharsany

**Affiliations:** 1 Health Economics and HIV and AIDS Research Division (HEARD), University of KwaZulu-Natal, Durban, South Africa; 2 Biostatistics Research Unit, South African Medical Research Council (SAMRC), Durban, South Africa; 3 Epicentre AIDS Risk Management (Pty) Limited, Durban, South Africa; 4 Centre for the AIDS Programme of Research in South Africa (CAPRISA), University of KwaZulu-Natal, Durban, South Africa; 5 School of Laboratory Medicine & Medical Sciences, Nelson R Mandela School of Medicine, University of KwaZulu-Natal, Durban, South Africa

## Abstract

**Background:**

HIV incidence among adolescent girls and young women (AGYW) remains high, with their male partners a prominent factor in sustaining these elevated rates. Partnership characteristics remain important metrics for determining HIV risk, with evidence indicating that AGYW engaged in transactional and age-disparate relationships face greater HIV exposure. This study examines the risk posed to AGYW in a relationship with a “Blesser”, defined as male who provides his female partner with their material needs or desires in exchange for a sexual relationship, an age-disparate (5 or more years older) partner, and the potential compounded risk of being a relationship with a partner or partners who are considered both a “Blesser” and age-disparate.

**Methods:**

A cross -sectional household based representative sample of AGYW (aged between 12–24 years) were enrolled in the study (*n* = 18 926) from the districts of City of Johannesburg and Ekurhuleni in the Gauteng province and the Districts of eThekwini and uMgungundlovu in the province of KwaZulu-Natal (KZN) in South Africa between March 13, 2017 to June 22, 2018. Participants completed a structured questionnaire and provided finger-prick blood samples for laboratory measurements. Our analysis used descriptive statistics and multiple binary logistic regressions accounting for survey weights, clustering and stratification.

**Findings:**

The median age of the sample was 21 years old (Interquartile range: 19–23) and nearly three quarters (73.7%) were currently attending school. Whilst all relationships exposed AGYW to potential HIV risk, multiple binary logistic regression analysis revealed that AGYW in a relationship with both a Blesser and an age-disparate partner were more likely to be HIV positive (AOR: 3.12, 95% CI: 1.76–5.53, *p* < 0.001), diagnosed with an STI (AOR: 4.60, 95% CI: 2.99–7.08, *p* < 0.001), had 2 or more sexual partners in the previous 12 months (AOR: 6.37, 95% CI: 3.85–10.54, *p* < 0.001), engaged in sexual activity at age 15 or younger (AOR: 3.67, 95% CI: 2.36–5.69, *p* < 0.001) and more likely to have ever been pregnant (AOR: 2.60, 95% CI: 1.24–5.45, *p* < 0.05) than those not in a relationship with either a Blesser or age-disparate partner.

**Conclusion:**

Different relationships present different HIV risk to AGYW. AGYW who had engaged in relationships with both a Blesser and an age-disparate partner were at greater HIV risk when examined against these relationships independent of one another. The data reveals the compounded HIV risk of being in both a transactional and age-disparate relationship.

## Introduction

Adolescent girls and young women (AGYW) aged 15–24 years remain highly susceptible to HIV acquisition, with this sub-population accounting for 29% of all new HIV infections in South Africa in 2018 (UNAIDS, 2020). There remains a number of biological, structural and behavioural factors which contribute to these high HIV rates [1]. This cocktail of factors play a significant role in increasing AGYW susceptibility to HIV infection. Structural factors, including low education levels, poverty and sexual abuse mediate many behavioural factors, including engaging in early sexual debut, transactional sex, age-disparate sex and multiple partnerships [2].

Epidemiological studies have demonstrated that both transactional sex and age-disparate relationships (ADR) increase HIV risk, particularly for AGYW [see 3, 4–11]. These risk behaviours are not mutually exclusive, with transactional sex having previously been reported within ADR [12]. Further, transactional sex can take a number of forms, shaped by a number of economic and social conditions. Literature suggests that transactional sex can be motivated by either ‘survival’ or ‘consumption’ [13]. Within the South African context, the economic and social conditions exist for transactional sex to be motivated by both ‘survival’ and in pursuit of social status or ‘consumption’. With respect to the latter, the ‘Blessed relationship’ has emerged, a convergence of technology, sexuality, and economics within a consumerist environment [14]. These relationships are characterised by young women seeking out men – known as ‘Blessers’ – who are willing and able to satiate their material desires in exchange for a sexual relationship [15]. Whilst ‘Blesser’ and ‘sugar daddy’ have been used interchangeably, literature has explained how blessed relationships have become a new South African cultural option of structuring relationships. The motivation for a ‘luxury’ lifestyle versus a relationship motivated by survival, is the defining feature of these blessed relationships [15]. Additionally, age disparity is not a prerequisite for blessed relationships, whereas sugar relationships are traditionally cross-generational [14].

Whilst blessed relationships remain transactional in nature [15], it is situated within the “sex for improved social status” paradigm, which Stoebenau (2016) argues places women as sexual agents who engage in this form of transactional sex for lifestyle attainment [16, 17]. Literature has however pointed out that despite these relationships being driven by aspiration as opposed to survival, women’s agency remains compromised [15], thereby potentially exposing women to risky sexual behaviour and increased HIV risk.

Literature has indicated that different relationship types are associated with HIV risk. This includes relationships that are transactional and age-disparate. Whilst research has identified that some ADR are transactional [12], there remains no epidemiological studies examining the relative risk of these relationships across the same population cohort, and the compounded risk for AGYW engaging in both transactional and ADR. Given that a Blessed relationship is transactional in nature, it can therefore be assumed that a relationship with a Blesser poses HIV risk to the female partner, despite a lack of data to prove this hypothesis. This study examines the HIV risk to AGYW who have self-identified as engaging in different relationship types across four districts in South Africa.

## Methods

### Study setting

Data were collected from the districts of City of Johannesburg and Ekurhuleni in the Gauteng province (GP) and the Districts of eThekwini and uMgungundlovu in the province of KwaZulu-Natal (KZN) in South Africa, from March 13, 2017 to June 22, 2018. The City of Ekurhuleni and City of Johannesburg districts are densely populated districts in the Gauteng Province, whilst both districts are urbanised having a diverse economy and large urban townships. eThekwini is the third most populous city in South Africa and the the largest city in the province of KwaZulu-Natal. eThekwini is home to the busiest port on the African continent and is the main economic hub within the province of KwaZulu-Natal. uMgungundlovu is the second largest district in KwaZulu-Natal and is located in central KwaZulu-Natal. It includes traditional settlements, farmlands, informal, urban and rural settlements. More detail on the districts, including the DREAMS programme, that the data were collected from has previously been described elsewhere [18].

### Study design

A cross-sectional household based survey targeting AGYW aged between 12 and 24 years old was conducted. Using a multistage cluster sampling method, four districts were selected as the primary strata. The sample size per district was allocated proportionately to the estimated number of AGYW in DREAMS sub–districts. Only small area layers (SAL) where the DREAMS programme was present were included in the sampling frame. SALs were included proportional to size from the sampling frame. Households within the SALs were randomly selected and all AGYW living in the household that met the requirements for study inclusion were enrolled. For this study we included AGYW who self-reported to be sexually active which was 44.5% (*n* = 8415) of the full sample.

### Data collection

Household information forms were administered to heads of the household and once it was confirmed that there were AGYW in the household who met the enrolment criteria, they were enrolled into the study. AGYW who were aged 12 to 24 years old, who were willing to participate, were legally able to provide written informed consent, and agreed to provide biological samples were included in the study. Both a caregiver and AGYW questionnaire were completed.

### Measures

Relationships were categorised into four types; 1) an ADR only (AGYW was 5 or more years younger than one of their male partners); 2) a relationship with both an age disparate partner and a Blesser (self-determined). Respondents selecting both could be referring to a single partner who was considered both a Blesser and who was age-disparate, or two or more partners who met this definition; 3) in a relationship with a Blesser only, and 4) all women who were sexually active but did not declare being in an relationship with a Blesser or age-disparate partner in the preceding 12 months. HIV status was ascertained through finger-prick blood samples into a BD Microtainer® (Becton Dickinson, South Africa) blood collection tubes for laboratory measurements of HIV antibodies. To determine whether respondents had recently had a sexually transmitted infection (STI) AGYW were asked if a health care professional had notified them of a STI in the previous 12 months. We also included three sexual risk variables; condom use in the previous 12 months was measured by asking respondents if they used condoms always, sometimes or never. We asked how many sexual partners they had in the previous 12 months, this was coded as 0 to 1 sex partners and 2 or more sex partners. We asked how old they were when they had vaginal or anal sex for the first time, this was coded as age 15 and younger or 16 and older. We asked if they are currently in school, no was coded as 0 and yes as 1. Pregnancy was measured by asking AGYW if they have ever been pregnant, no was coded as 0 and yes as 1. We asked respondents their marital status, including whether they were married and whether they were co-habitating. Food security was measured by asking if in the past four weeks, how often any member of their household went to sleep hungry, with response options including always, often, sometimes and never.

### Data analysis

Data analysis was undertaken using SPSS version 27. To adjust for the study design and non-response, sampling weights were used. The final individual weights were benchmarked against the 2018 Statistics South Africa mid-year population estimates by age group and province [19]. Taylor series linearization methods were used to estimate standard errors. In all analyses, standard errors were adjusted for stratification by district and clustering by SAL. Our analysis used descriptive statistics (unweighted counts and weighted percentages) and multiple binary logistic regressions. We assessed the relationship between HIV status and relationship type, STI status and sexual risk behaviours while controlling for age and food security. Adjusted Odds Ratios (AOR) with 95% confidence intervals (CI) for the multiple logistic regressions were calculated.

## Results

The median age for the study sample (*n* = 8415) was 21 (IQ: 19–23) years (see Table 1). Just over two-thirds (69.5%) of the sample were 20–24 years old. Only 2.0% of the respondents indicated they were married or engaged. The vast majority (78.1%) were in a non-cohabitating relationship. The majority of respondents engaged in risky sexual behaviour with 79.4% using condoms inconsistently in the previous 12 months and just over half (56.1%) having had two or more sexual partners in the previous 12 months. The HIV prevalence was 5.7% among this sample of AGYW, whilst 8.7% self-reported having had a STI in the previous 12 months.

**Table 1:** Weighted percentages and unweighted counts for the four relationship types by sociodemographic, sexual risk, HIV status and STI status variables

Variable		Only Blessed		Blessed + ADR		Only ADR		Not blessed or ADR		Total	
		N	%	n	%	n	%	n	%	n	%
Age categories	12–14 yrs	1	1.2%	0	0.0%	25	0.8%	61	0.9%	87	0.8%
	15–19 yrs	28	29.8%	37	27.5%	676	23.9%	2041	32.4%	2782	29.7%
	20–24 yrs	57	69.0%	87	72.5%	1773	75.4%	3629	66.8%	5546	69.5%
Median age (IQR)		86	21(19–23)	124	21 (19–22)	2474	21 (20–23)	5731	21 (19–22)	8415	21 (19–23)
Currently attending school	No	8	36.1%	10	39.9%	155	33.4%	346	23.6%	519	26.3%
	Yes	13	63.9%	16	60.1%	295	66.6%	1041	76.4%	1365	73.7%
In the past 4 weeks, how often go to sleep hungry	Often	2	1.9%	4	2.8%	82	3.4%	170	3.1%	258	3.1%
	Sometimes	12	14.3%	16	14.3%	246	10.5%	507	8.9%	781	9.5%
	Rarely	5	5.3%	20	15.1%	171	7.2%	458	8.4%	654	8.1%
	Never	59	68.7%	72	59.1%	1780	72.3%	3982	69.9%	5893	70.4%
	Missing data	8	9.8%	12	8.6%	195	6.6%	614	9.8%	829	8.8%
Relationship status	Single not cohabiting	14	15.4%	14	13.0%	263	11.2%	858	15.2%	1149	14.0%
	In a relationship and not cohabiting	67	78.1%	98	76.5%	1884	74.3%	4526	77.9%	6575	76.8%
	Unmarried and cohabiting	3	3.4%	12	10.5%	252	11.2%	270	5.4%	537	7.2%
	Engaged/married	2	3.1%	0	0.0%	72	3.1%	72	1.4%	146	2.0%
	Widow/divorced/separated	0	0.0%	0	0.0%	3	0.1%	5	0.1%	8	0.1%
Condom use previous 12 months	Consistent use	19	23.0%	22	17.2%	393	16.8%	1201	22.3%	1635	20.6%
	Inconsistent use	67	77.0%	102	82.8%	2081	83.2%	4417	77.7%	6667	79.4%
Number sex partners in prev. 12 mo	0–1 partners	31	35.3%	21	14.1%	806	31.8%	2864	50.2%	3722	43.9%
	2 + partners	55	64.7%	103	85.9%	1668	68.2%	2754	49.8%	4580	56.1%
Sexual debut age	16 + years old	66	77.4%	80	65.2%	1902	81.0%	4576	85.0%	6624	83.4%
	= < 15 years old	20	22.6%	44	34.8%	490	19.0%	856	15.0%	1410	16.6%
HIV status	Negative	78	91.2%	108	86.9%	2259	92.2%	5452	95.4%	7897	94.3%
	Positive	8	8.8%	16	13.1%	215	7.8%	279	4.6%	518	5.7%
Been told they have an STI	No	74	87.9%	90	73.2%	2112	89.1%	4987	92.8%	7263	91.3%
	Yes	11	12.1%	30	26.8%	260	10.9%	386	7.2%	687	8.7%

*Notes*: Only ADR (AGYW was 5 or more years younger than one of their male sex partners); a relationship with both an age-disparate partner and a Blesser; in a relationship with a Blesser only, and the last category included all women who were sexually active but did not declare being in an relationship with a Blesser or age-disparate partner

The descriptive statistics were disaggregated according to relationship categories; relationship with a Blesser only (*n* = 86, 1% of total sample); relationship with Blesser and age-disparate partner (*n* = 124, 1.5% of total sample); Age-disparate partner only (*n* = 2474, 29.4% of total sample) and sexually active AGYW who declared they hadn’t been in a relationship with a Blesser or age-disparate partner in the previous 12 months (*n* = 5731, 68.1% of total sample). The median age was the same in each relationship category (21 years old). AGYW not in a relationship with a Blesser or age-disparate partner were more likely to be attending school (76.4%) compared with AGYW engaged in a relationship with a Blesser (63.9%), age-disparate partner (66.6%) and an age-disparate partner and Blesser (60.1%). AGYW who were in a relationship with both a Blesser and an age disparate partner were the most likely (85.9%) of the sample of AGYW to have had 2 or more partners in the previous 12 months and were also more likely (34.8%) to have sexually debuted at 15 years or younger. HIV and STI prevalence were highest (13.1% & 26.8%) amongst AGYW who had been in a relationship with a Blesser or age-disparate partner (see Table 1).

Table 2 shows the multiple binary logistic regression analysis between the different forms of relationships and risk behaviours and HIV and STI prevalence. Those who were in a relationship with a Blesser and an age-disparate partner were more likely to be HIV positive (AOR: 3.12, 95% CI: 1.76–5.53, *p* < 0.001), diagnosed with an STI (AOR: 4.60, 95% CI: 2.99–7.08, *p* < 0.001), had 2 or more sexual partners in the previous 12 (AOR: 6.37, 95% CI: 3.85–10.54, *p* < 0.001), engaged in sexual activity at age 15 or younger (AOR: 3.67, 95% CI: 2.36–5.69, *p* < 0.001) and more likely to have ever been pregnant (AOR: 2.60, 95% CI: 1.24–5.45, *p* < 0.05) when compared to those not in a relationship with either a Blesser or age-disparate partner. AGYW who were only in an age disparate relationship were more likely to be HIV positive (AOR: 1.62, 95% CI: 1.33–1.97, *p* < 0.001) and more likely to have 2 or more sexual partners in the previous 12 months (AOR: 2.07, 95% CI: 2.07, *p* < 0.001) when compared to those not in a relationship with either a Blesser or age-disparate partner.

**Table 2:** Multiple logistic regression analysis for AGYW not in a blessed or age-disparate relationship compared with the three other types of relationships and controlling for age and food security

	HIV	STI	Condom use	Number of sex partners	Sexual debut age	School attendance^a^	Pregnancy^a^
	AOR (95% CI)	AOR (95% CI)	AOR (95% CI)	AOR (95% CI)	AOR (95% CI)	AOR (95% CI)	AOR (95% CI)
Not in blessed or ADR	Ref	Ref	Ref	Ref	Ref	Ref	Ref
Only Blesser	1.93 (0.83–4.49)	1.75 (0.87–3.53)	0.96 (0.55–1.65)	1.85* (1.12–3.07)	1.84* (1.03–3.30)	0.50 (0.17–1.49)	1.22 (0.53–2.78)
Blesser and age-disp. par	3.12*** (1.76–5.53)	4.60*** (2.99–7.08)	1.38 (0.84–2.27)	6.37*** (3.85–10.54)	3.67*** (2.36–5.69)	0.42 (0.17–1.02)	2.60* (1.24–5.45)
Only ADR	1.62*** (1.33–1.97)	1.52*** (1.27–1.83)	1.36*** (1.19–1.56)	2.07*** (1.85–2.32)	1.66*** (1.44–1.92)	0.61*** (0.47–0.79)	1.49*** (1.23–1.79)
Food insecure (Often)	1.44 (0.72–2.89)	1.34 (0.77–2.32)	0.79 (0.54–1.15)	1.01 (0.71–1.44)	0.41*** (0.27–0.61)	0.25** (0.11–0.60)	0.84 (0.45–1.54)
Food insecure (Sometimes)	1.15 (0.60–2.19)	1.11 (0.69–1.78)	1.05 (0.79–1.39)	1.55** (1.21–2.00)	0.36*** (0.26–0.49)	0.38** (0.21–0.68)	1.42 (0.92–2.20)
Food insecure (Rarely)	0.98 (0.50–1.89)	1.22 (0.76–1.95)	1.22 (0.88–1.69)	1.65*** (1.25–2.17)	0.30*** (0.22–0.42)	0.29*** (0.15–0.56)	0.91 (0.57–1.47)
Food insecure (Never)	0.94 (0.53–1.68)	0.98 (0.66–1.47)	1.16 (0.92–1.47)	1.47*** (1.20–1.80)	0.25*** (0.19–0.32)	0.32*** (0.19–0.52)	0.99 (0.69–1.44)
Food insecure (Missing data)	Ref	Ref	Ref	Ref	Ref	Ref	Ref
Age (years)	1.21*** (1.14–1.28)	1.07** (1.03–1.12)	1.10*** (1.06–1.14)	1.13*** (1.10–1.16)	0.82*** (0.79–0.85)	0.81 (0.63–1.04)	1.38*** (1.21–1.56)
Pseudo R-squared	0.040	0.024	0.024	0.088	0.177	0.149	0.064
N	8415	7950	8302	8302	8034	1884	2720

*Notes*: ^a^ restricted to those 19 years old or younger. * *p* < 0.01 ** *p* < 0.01 *** *p* < 0.001

The results from the regression analysis comparing those in a relationship with a Blesser and an age-disparate partner with those only in an ADR can be found in Table 3. These reveal that AGYW in both a relationship with a Blesser and an age-disparate partner were more likely to: be HIV positive (AOR: 1.93, 95% CI: 1.08–3.46, *p* < 0.05); diagnosed with an a STI (AOR: 3.02, 95% CI: 1.94–4.72, *p* < 0.001); had a higher number of sex partners (AOR: 3.08, 95% CI: 1.85–5.14, *p* < 0.05); and sexually debuted at a younger age (AOR: 2.20, 95% CI: 1.42–3.43, *p* < 0.001). Those not in a blessed or ADR were less likely to be HIV positive (AOR: 0.62, 95% CI: 0.51–0.76, *p* < 0.001); diagnosed with an STI (AOR: 0.66, 95% CI: 0.55–0.79, *p* < 0.001); use a condom inconsistently (AOR: 0.74, 95% CI: 0.64–0.84, *p* < 0.001), have 2 or more sex partners (AOR: 0.48, 95% CI: 0.43–0.54, *p* < 0.001); engage in early sexual debut (AOR: 0.60, 95% CI: 0.52–0.70, *p* < 0.001); and less likely to be pregnant (AOR: 0.67, 95% CI: 0.56–0.81, *p* < 0.001) than those who were in only an age disparate relationship. Those who were not in a Blessed or ADR were more likely to be currently attending school than those only in an ADR (AOR: 1.65, 95% CI: 1.27–2.15, *p* < 0.01).

**Table 3:** Multiple logistic regression analysis for AGYW in an ADR only compared with the three other types of relationships and controlling for age and food security

	HIV	STI	Condom use	Number of sex partners	Sexual debut age	School attendance^a^	Pregnancy^a^
	AOR (95% CI)	AOR (95% CI)	AOR (95% CI)	AOR (95% CI)	AOR (95% CI)	AOR (95% CI)	AOR (95% CI)
Only ADR	Ref	Ref	Ref	Ref	Ref	Ref	Ref
Only Blesser	1.20 (0.51–2.78)	1.15 (0.57–2.33)	0.70 (0.40–1.23)	0.90 (0.54–1.49)	1.11 (0.61–2.02)	0.83 (0.28–2.47)	0.82 (0.35–1.90)
Blesser and age-disp. par	1.93* (1.08–3.46)	3.02*** (1.94–4.72)	1.01 (0.60–1.70)	3.08*** (1.85–5.14)	2.20*** (1.42–3.43)	0.69 (0.28–1.73)	1.75 (0.82–3.71)
Not in blessed or ADR	0.62*** (0.51–0.76)	0.66*** (0.55–0.79)	0.74*** (0.64–0.84)	0.48*** (0.43–0.54)	0.60*** (0.52–0.70)	1.65** (1.27–2.15)	0.67*** (0.56–0.81)
Food insecure (Often)	1.44 (0.72–2.89)	1.34 (0.77–2.32)	0.79 (0.54–1.15)	1.01 (0.71–1.44)	0.41*** (0.27–0.61)	0.25** (0.11–0.60)	0.84 (0.45–1.54)
Food insecure (Sometimes)	1.15 (0.60–2.19)	1.11 (0.69–1.78)	1.05 (0.79–1.39)	1.55** (1.21–2.00)	0.36*** (0.26–0.49)	0.38** (0.21–0.68)	1.42 (0.92–2.20)
Food insecure (Rarely)	0.98 (0.50–1.89)	1.22 (0.76–1.95)	1.22 (0.88–1.69)	1.65*** (1.25–2.17)	0.30*** (0.22–0.42)	0.29*** (0.15–0.56)	0.91 (0.57–1.47)
Food insecure (Never)	0.94 (0.53–1.68)	0.98 (0.66–1.47)	1.16 (0.92–1.47)	1.47*** (1.20–1.80)	0.25*** (0.19–0.32)	0.32*** (0.19–0.52)	0.99 (0.69–1.44)
Food insecure (Missing data)	Ref	Ref	Ref	Ref	Ref	Ref	Ref
Age	1.21*** (1.14–1.28)	1.07** (1.03–1.12)	1.10*** (1.06–1.14)	1.13*** (1.10–1.16)	0.82*** (0.79–0.85)	0.81 (0.63–1.04)	1.38*** (1.21–1.56)
Pseudo R-squared	0.040	0.024	0.024	0.088	0.177	0.149	0.064
N	8415	7950	8302	8302	8034	1884	2720

^a^ Restricted to those 19 years old or younger. * *p* < 0.01 ** *p* < 0.01 *** *p* < 0.001

## Discussion

This study examined the HIV risk for sexually active AGYW across a number of partnership types in a high HIV prevalence setting in South Africa. Our data, which reveals the increase in HIV risk among AGYW engaged in ADRs, is in keeping with extent literature [6, 9, 11, 12]. These data further reveal the elevated HIV risk for AGYW engaged in relationships with a Blesser. Whilst there is no previous epidemiological data examining the risk posed specifically by Blessers, other studies have shown that transactional sex is associated with increased HIV incidence and prevalence among young women in similar contexts [20, 21]. Of concern, this effect has been shown to be driven by relationships in which sexual partners provide money and/or gifts on a frequent, more regular basis, indicating that the inherent risk of transactional sex for HIV increases with the frequency by which young women receive material items from their partners [22]. The hypothesis here is that the frequency of gift giving is associated with an increase in coital frequency [22]. Given that blessed relationships are distinguished by their longevity and maintained through the satiation of the needs of both the women, material and lifestyle, and the male partner, companionship and sex, our data supports this hypothesis.

HIV risk is compounded when AGYW are in a relationship with both a Blesser and age-disparate partner. HIV prevalence and STI diagnosis were highest amongst AGYW in, or having been in a relationship with someone or multiple partners considered both a Blesser and age-disparate. Key high risk behaviours were also elevated within this group, with early sexual debut and multiple partners pronounced characteristics of AGYW engaging with both partnership types. Age-disparate partners increase the exposure of AGYW to infection due to them being in a higher HIV prevalence pool [23], whilst relationships with Blessers presents an unequal power dynamic as characterised in other transactional relationships, especially where AGYW come from poor households [24]. Even in instances where AGYW do not come from particularly difficult economic circumstances, the motivation exists to cede to the sexual demands of the Blesser in order to sustain the relationship [15]. AGYM are having to navigate the duality of harm-benefit which exists within these relationships, with previous research already calling for interventions that address the motivation, and accounting for realities faced by AGYW within these contexts [25]. Our findings further reveal that a higher proportion of AGYW in a relationship with either a Blesser or age-disparate partner were not attending school. Previous literature has shown that increased school attendance among AGYW is associated with a reduction in risky sexual behaviour [23, 26] and HIV incidence [27, 28]. School retention remains a key intervention aimed at reducing HIV risk amongst AGYW.

This study further indicates the relative risk for AGYW in a relationship with an age disparate partner only, compared with AGYW in a relationship with a partner or multiples partners considered a Blesser and age-disparate. The latter characterised by higher rates of HIV and other STIs, and higher proportion who had engaged in sexual activity by age 15.

This study has several limitations. Research has found that sensitive behavioural and STI data are not accurately reported [29], however we used categories instead of continuous variables which should help to reduce some of this bias. The cross-sectional nature of these data makes it difficult to interpret causal relationships between the independent and dependent variables. Longitudinal data are needed to assess the influence of relationship status on HIV and STI status. The study selected only sexually active participants from DREAMS programme areas, therefore the results are not representative of the KZN and GP or the four districts. Participants were asked whether they had engaged in a sexual relationship with a Blesser and an age-disparate partner separately. We cannot determine whether those who answered in the affirmative for both were referring to the same or different individuals. We further relied on respondents own understanding of the term Blesser. Whilst the term remains prevalent within the broader South African context, some respondents may not have recognised the term.

## Conclusion

Relationship type has shown to make a significant difference to the HIV risk presented to AGYW. AGYW in a relationship with a partner considered both a Blesser and age-disparate were at greater risk when compared against other partnership types separately. AGYW need to understand the inherent risk posed by their sexual choices. Interventions need to account for both the current and evolving contextual factors affecting AGYW sexual behaviour.

## Data Availability

The dataset analysed for the current study is available in the Figshare repository, 10.6084/m9.figshare.19096250.
